# *Bifidobacterium* and *Lactobacillus* Composition at Species Level and Gut Microbiota Diversity in Infants before 6 Weeks

**DOI:** 10.3390/ijms20133306

**Published:** 2019-07-05

**Authors:** Bo Yang, Yingqi Chen, Catherine Stanton, R. Paul Ross, Yuan-Kun Lee, Jianxin Zhao, Hao Zhang, Wei Chen

**Affiliations:** 1 State Key Laboratory of Food Science and Technology, Jiangnan University, Wuxi 214122, China; 2 School of Food Science and Technology, Jiangnan University, Wuxi 214122, China; 3 International Joint Research Center for Probiotics & Gut Health, Jiangnan University, Wuxi 214122, China; 4 Teagasc Food Research Centre, Moorepark, Fermoy, P61 C996 Co. Cork, Ireland; 5 APC Microbiome Ireland, University College Cork, T12 K8AF Cork, Ireland; 6 Department of Microbiology and Immunology, National University of Singapore, Singapore 117545, Singapore; 7 National Engineering Research Center for Functional Food, Jiangnan University, Wuxi 214122, China; 8 Beijing Innovation Center of Food Nutrition and Human Health, Beijing Technology and Business University (BTBU), Beijing 102488, China

**Keywords:** gut microbiota, *Bifidobacterium* communities, *Lactobacillus* communities, diversity, infants, functional prediction

## Abstract

Our objective was to investigate the effects of different delivery and feeding modes on the gut microbiota composition of early infants with special emphasis on *Bifidobacterium* and *Lactobacillus* profiles at species level. 16S rRNA V3-V4 regions, bifidobacterial, and lactobacilli *groEL* genes from infant feces were sequenced by Illumina MiSeq. Gut microbiota abundance was significantly different, where standard vaginally delivered (SVD) and breast-fed (BF) groups were higher in comparison with caesarean section (CS), milk-powder-fed (MPF), and mixed-fed (MF) groups. The genus unclassified Enterobacteriaceae was dominant, followed by *Bifidobacterium*, which was highly abundant in SVD and BF groups. The dominant *Bifidobacterium* species in all groups were *B. longum subsp. longum*, *B. longum subsp. infantis* and *B. animalis subsp. lactis*. *B. dentium* and the diversity of *Bifidobacterium* in SVD and BF groups were significantly higher. For *Lactobacillus* profiles, *L. rhamnosus* and *L. gasseri* were dominant among all the groups, while *Lactobacillus* species in CS and MPF groups were more diverse. Functional predictions showed significant differences between delivery mode and feeding groups, such as phosphotransferase system as well as taurine and hypotaurine metabolism. In early infants with different delivery and feeding methods, gut microbiota—particularly bifidobacteria and lactobacilli communities—showed significant differences, with strong implications for physiological functions.

## 1. Introduction

There are approximately 10^14^ microorganisms in the human gut, encoding 100 times more genes than the human genome [1]. Many studies have shown that gut microbiota play important roles in human health and wellbeing [2]. The colonization of gut microbiota in infants is a critical period for gut microbiota formation and maturity, affecting future growth and immune system development [3]. Moreover, *Bifidobacterium* is one of the dominant bacterial genera, and has important effects on the development of gut microbiota during early and subsequent infant physiological state and health [4]. There is ample evidence that *Bifidobacterium* and *Lactobacillus* supplementation have positive effects on the protection of human gut from different intestinal infections [5], and they are associated with the production of beneficial metabolites [6]. 

The structure and composition of infant gut microbiota are affected by many factors, including genetic factors and the intra-fetal environment, while antibiotics, diet, and probiotics influence microbiota development [7]. Recent studies have shown that delivery mode and feeding type are important factors influencing gut microbiota, and have significant correlation with the intestinal microbial diversity of infants at 6 weeks of age [8]. Mode of delivery (e.g., standard vaginally delivered (SVD) and caesarean section (CS)) have been shown to affect the colonization of gut microbiota in neonates and infants—especially the number and composition of *Bifidobacterium* and *Lactobacillus* [9]. The microbes in the gut of SVD newborns are similar to those in the maternal skin and vagina, with *Enterococcus*, *Streptococcus*, *Lactobacillus*, *Clostridium*, and *Bifidobacterium* found to be prevalent [10], while skin bacteria and bacteria in the hospital environment have been found to colonize the gut of CS newborns, leading to low abundance of *Bifidobacterium* and lower bacterial diversity, and have been suggested to destroy the normal establishment of infant gut microbiota [11]. 

Among feeding methods, infants can be breast-fed (BF), milk-powder-fed (MPF), or mixed-fed (MF), which have significant differential influences on gut microbiota. During breastfeeding, the digestive tract is colonized by Actinobacteria and Firmicutes. The *Actinobacteria* are mainly composed of *Bifidobacterium*, which contains *B. breve*, *B. longum subsp. longum*, *B. bifidum*, *B. longum subsp. infantis*, and *B. pseudocatenulatum* [12], and *Firmicutes* are composed of *Lactobacillus* (*L. rhamnosus*, *L. gasseri*), *Enterococcus*, *Clostridium*, etc. [13]. Healthy mothers’ breast milk contains ~10^9^ microbes per liter and is considered the perfect source of nutrition for infants [14]. Therefore, breastfeeding is critical for the establishment of gut microbiota.

In general, delivery mode and feeding regimen are important factors affecting infants’ gut microbiota. Some studies have also shown that SVD and BF were more conducive to the colonization and development of healthy infant intestinal microbes [15]. However, the functionality of the respective bacterial species—in particular *Bifidobacterium* and *Lactobacillus* species which contribute to the health status of infants—has not been systematically studied and verified. The aim of the current study was to investigate the gut microbiota in infants within 6 weeks of birth in China via high-throughput sequencing technology, focusing on the bacterial diversity, composition, and functionality of bifidobacteria and lactobacilli at the species level, to evaluate the effects of delivery mode and feeding method. 

## 2. Results

### 2.1. Gut Microbiota Composition in Infants

**Figure 1:**
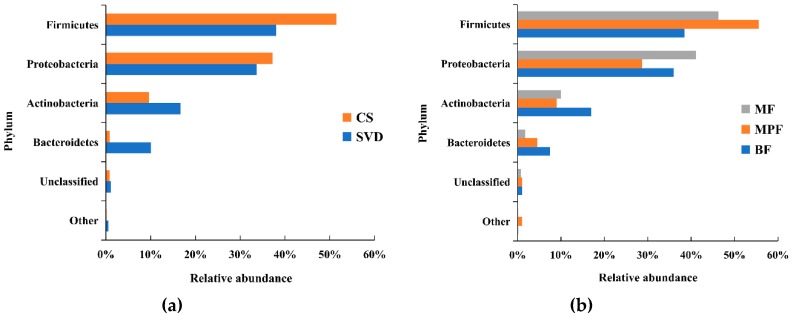
Composition of infant gut microbiota at the phylum level in different delivery modes and feeding methods. (**a**) Delivery modes; (**b**) Feeding methods. Showing phyla found at >1% average in total population. Phyla found at <1% were grouped as “other”. BF: breast-fed; CS: caesarean section; MF: mixed-fed; MPF: milk-powder-fed; SVD: standard vaginally delivered.

In total, 23 phyla were detected by Illumina sequencing of the V3–V4 region in all samples, but 98.3% of the sequences among all samples were mainly composed of four phyla, including Firmicutes, Proteobacteria, Actinobacteria, and Bacteroidetes. Firmicutes and Proteobacteria were the dominant phylum. However, statistical analysis showed only a significant difference in Bacteroidetes based on delivery mode (*p* < 0.01), and the differences between the four phyla in the feeding groups were not significant (*p* > 0.05). Compared the two different delivery modes (Figure 1a), the abundance of Firmicutes in the CS group (51.47%) was higher than that in SVD group (37.98%), while in the SVD group there were more Actinobacteria (mainly comprised of *Bifidobacterium*) and Bacteroidetes than in the CS group.

Among the different feeding methods (Figure 1b), the abundance of Firmicutes in the MPF group (55.56%) was significantly higher than that in both BF (38.50%) and MF (46.26%) groups, while the abundance of Proteobacteria (28.72%) was significantly lower than in the other two groups (i.e., 41.14% and 36.00%). In the BF group, the abundance of Actinobacteria and Bacteroidetes were both higher than in the other two groups.

**Figure 2:**
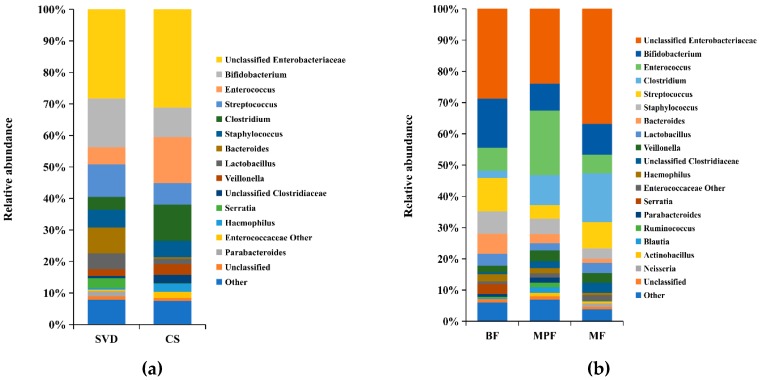
Composition of infant gut microbiota at the genus level in different delivery modes and feeding methods. (**a**) Delivery modes; (**b**) Feeding methods. Showing genera found at >1% average in total population. Genera found at <1% were grouped as “other”.

Analysis of the composition of gut microbiota in different delivery modes showed that more than 60% of the genera were unclassified Enterobacteriaceae (28.26%), *Bifidobacterium* (15.45%), *Streptococcus* (10.30%) and *Bacteroides* (8.13%) in the SVD group, while unclassified Enterobacteriaceae (31.13%), *Enterococcus* (14.68%), *Clostridium* (11.54%), and *Bifidobacterium* (9.36%) were the dominant genera in the CS group (Figure 2a). It showed that unclassified Enterobacteriaceae and *Bifidobacterium* were the common genera in both delivery modes, while the abundance of unclassified Enterobacteriaceae and *Bifidobacterium* had no significant difference (*p* > 0.05). Moreover, *Streptococcus* and *Bacteroides* were the dominant genera in SVD group, while *Enterococcus* and *Clostridium* were the specific dominant genera in the CS group, and the abundance of *Enterococcus* and *Clostridium* had significant differences between these two groups (*p* < 0.05). The statistical analysis of some low-abundance microbiota showed that *Bacteroides* (8.13%) in the SVD group was 19 times higher than in the CS group, and the difference was significant (*p* < 0.01), while the relative ratios of *Parabacteroides* and *Serratia* in SVD and CS groups were 39:1 and 68:1, which was significantly different (*p* < 0.05). 

The colony structure of gut microbiota in the infants with different feeding methods showed that unclassified Enterobacteriaceae (BF 28.71%, MPF 23.94%, MF 36.81%) was the main dominant genera in all three groups, followed by *Bifidobacterium* (BF 15.72%, MPF 8.67%, MF 9.89%) which was highest in the BF group (Figure 2b). The abundance of *Streptococcus* (10.73%) was relatively high in the BF group, while only *Enterococcus* (20.62%) was significantly higher in the MPF group and had significant difference among the three groups (*p* < 0.05). Additionally, *Clostridium* (15.72%) was highest in the MF group, and the difference was significant (*p* < 0.05). 

### 2.2. Gut Microbiota Diversity in Early Infants

To explore the effect of delivery mode and feeding regimen on the diversity of gut microbiota, the beta diversity among different samples was analyzed. The distribution of samples in different delivery modes showed that the CS group was separated from the SVD group (Figure S1), but they were clustered into different types. The distribution of samples under different feeding methods showed that BF and MPF groups could be clustered into two types (Figure S1), while some samples of the MF group were dispersed in the other two groups, and only half of the samples were grouped together.

**Figure 3:**
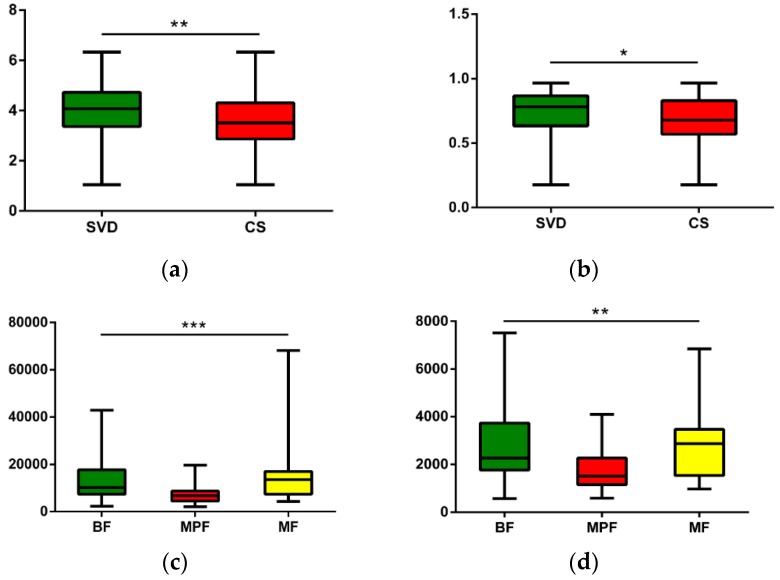
Alpha diversity analysis of infant gut microbiota in different delivery modes and feeding methods. (**a**) Delivery modes, Shannon (*p* < 0.01); (**b**) Delivery modes, Simpson (*p* < 0.05); (**c**) Feeding methods, Chao1 (*p* < 0.001); (**d**) Feeding methods, Observed species (*p* < 0.01). * *p* < 0.05, ** *p* < 0.01, *** *p* < 0.001.

The operational taxonomic units (OTUs) of each sample were used to calculate the alpha diversity for further validation. Four different alpha diversity indices were used to characterize bacterial abundance and community stability. The Shannon and Simpson indexes had significant differences in describing different delivery modes (Figure 3, Shannon Index: *p* < 0.01, Simpson: *p* < 0.05). Different feeding methods had notably significant differences in Chao1 and Observed Species (Figure 3, Chao1: *p* < 0.001, Observed Species: *p* < 0.01).

**Figure 4:**
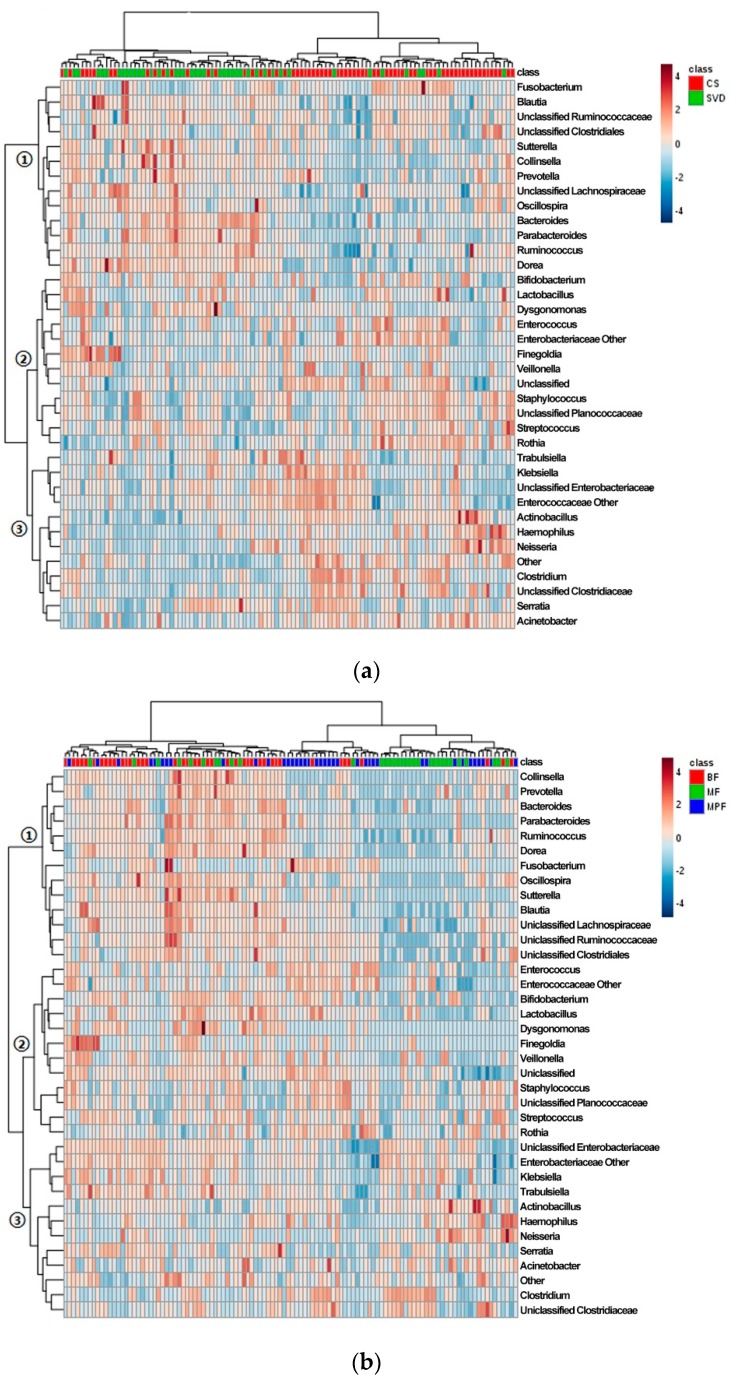
Cluster analysis of infant gut microbiota in different delivery modes and feeding methods. (**a**) Delivery modes; (**b**) Feeding methods. Only those genera (side) that were present in at least 1% 0f samples (top) were shown.

The cluster heat map of the horizontal abundance of V3V4 level (Figure 4a) showed that most samples were clustered according to delivery modes, and both CS and SVD groups showed relatively tight clusters. The abundant genera in the SVD group were relatively reduced in the CS group (branch 1), such as *Bacteroides* and *Parabacteriodes*, while the genera with relatively low abundance in the SVD group were higher in the CS group (branch 3), especially *Clostridium* and *Haemophilus*. Some species had little difference in abundance between the two groups (branch 2), such as *Bifidobacterium* and *Lactobacillus*. The genus-level abundance of the SVD group was slightly higher than that of the CS group. 

Most samples were clustered according to the feeding regimen (Figure 4b). BF and MPF groups showed a tighter cluster than MF group, which might be due to the mixed feeding including breast milk and formula. The most abundant genera in the BF group were extremely low in the other two groups (branch 1), such as *Bacteroides* and *Ruminococcus*, and the abundant genera in BF and MF groups were lower in the MPF group (branch 2), such as *Bifidobacterium* and *Lactobacillus*. Some genera in the three groups had similar abundance (branch 3), especially Enterobacteriaceae and *Clostridium*. The BF group had the highest genus-level abundance, followed by the MF group, and the MPF group was the lowest.

**Figure 5:**
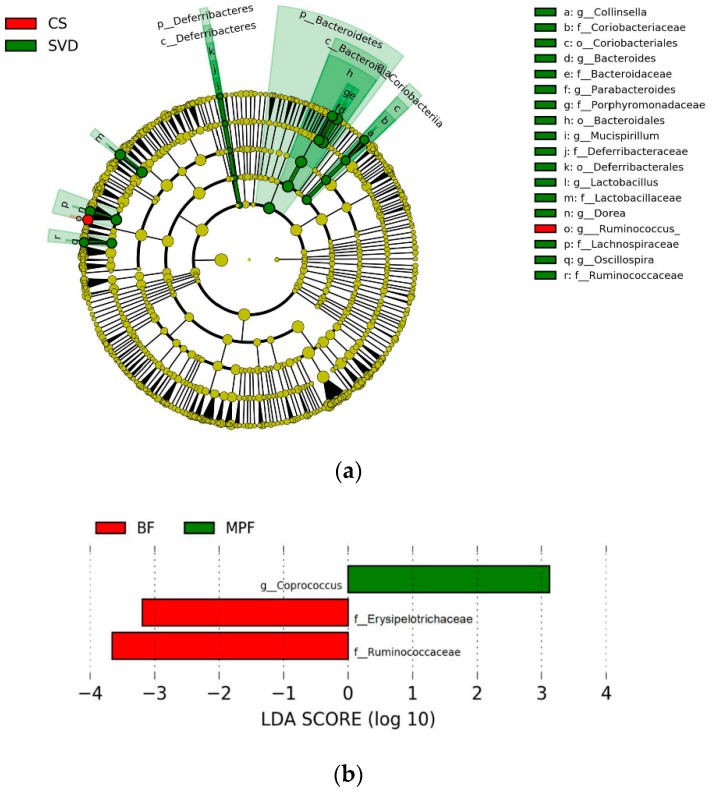
Linear discriminant analysis (LDA) effect size (LEfSe) analysis of infant gut microbiota in different delivery modes and feeding methods. (**a**) Delivery modes (alpha value = 0.01, LDA score = 3.0); (**b**) Feeding methods (alpha value = 0.05; LDA score = 2.0).

To investigate the communities or species that had significant differences in sample partitioning, the results were analyzed by linear discriminant analysis (LDA) effect size (LEfSe analysis), using linear judgment (LDA) to estimate the effect of the abundance of each component on the difference effect. In the different delivery modes, the microbiota showing a significant difference in the CS group was only *Ruminococcus*, while the SVD group had a large number of different microbiota, mainly Bacteroidaceae, Lactobacillaceae, Lachnospiraceae, Porphyromonadaceae, Deferribacteraceae, Ruminococcaceae, Coriobacteriaceae, etc. (Figure 5a, alpha value = 0.01, LDA score = 3.0). Similarly, among the different feeding regimens, only BF and MPF groups had significant microbiota differences, which were Ruminococcaceae, Erysipelotrichaceae, and *Coprococcus* (Figure 5b, alpha value = 0.05, LDA score = 2.0). 

### 2.3. Diversity of *Bifidobacterium* in Early Infant Gut

#### 2.3.1. Bifidobacterial Composition in Infants 

**Figure 6:**
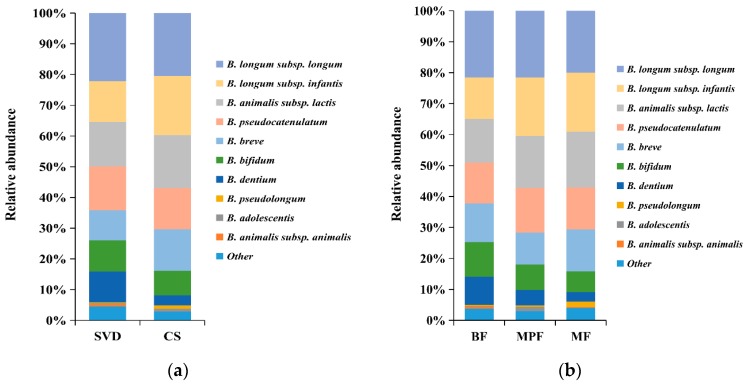
Bifidobacterial composition of infant guts in different delivery modes and feeding methods. (**a**) Delivery modes; (**b**) Feeding methods. Showing species found at >1% average in total population. Species found at <1% were grouped as “other”.

The difference in bifidobacterial composition in different delivery modes and feeding methods showed that the dominant species in SVD and CS groups were *B. longum* subsp. *longum*, *B. longum* subsp. *infantis*, *B. animalis* subsp. *lactis*, *B. pseudocatenulatum*, *B. breve*, and *B. bifidum*, which accounted for 80% and more than 90% of the total bifidobacteria communities, respectively (Figure 6a). In addition, the content of *B. dentium* in the SVD group (10.01%) was three times than that in the CS group. *B. animalis* subsp. *animalis*, *B. dentium*, *and B. longum* subsp. *infantis* had significant differences between the groups (*p* < 0.05). 

The above six bifidobacteria species were also dominant species in BF, MPF, and MF groups, accounting for 80%, 90%, and 90% of the total bifidobacteria, respectively (Figure 6b). Similarly, the content of *B. dentium* in the BF group (9.10%) was the highest (about twice that in the MPF group and three times that in the MF group, but not significantly different between MPF and MF groups). Only *B. adolescentis* showed a significant difference (*p* < 0.01) among these three groups. 

#### 2.3.2. Analysis of Bifidobacterial Diversity and Related Factors in Early Infants

To explore the differential effects of delivery mode and feeding methods on bifidobacteria, the diversity analysis of *Bifidobacterium* was performed at the species level. The distribution of bifidobacteria with different delivery modes were mostly aggregated and individually scattered, but the distribution of the CS group was more dense than the SVD group (Figure S2), and there was no obvious clustering between the two groups. The distribution of samples under different feeding methods showed that the MF group was relatively densely distributed (Figure S2), while BF and MPF groups were relatively sparse and similar in degree, and there was no significant clustering among the three groups.

The alpha diversity of samples was calculated based on the generated OTU results to deepen our investigation. The Observed Species and Shannon index showed that there was a significant difference in describing different feeding methods (Figure S3, Observed Species: *p* < 0.05, Shannon Index: *p* < 0.001), while in delivery mode, the *p*-values of all indexes were greater than 0.05, and therefore the difference was not significant.

### 2.4. Diversity of *Lactobacillus* in Early Infant Gut

#### 2.4.1. *Lactobacillus* Composition in Infants

**Figure 7:**
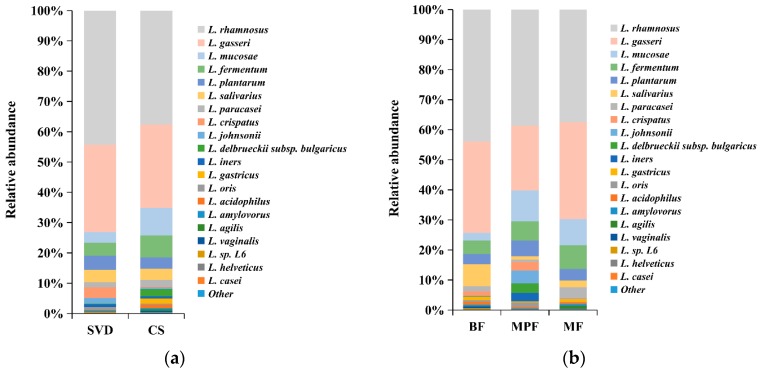
*Lactobacillus* composition of infant gut in different delivery modes and feeding methods. (**a**) Delivery modes; (**b**) Feeding methods. Showing species found at >1% average in the total population. Species found at <1% were grouped as “other”.

Although statistical analysis showed that the differences in the abundance of *Lactobacillus* between delivery mode and feeding groups were not significant, there were some differences in the species composition among different groups. The differences in the composition of *Lactobacillus* in different delivery modes and feeding methods showed that both SVD and CS groups were dominated by *L. rhamnosus* and *L. gasseri*, accounting for 70% and 60% of the total lactobacilli (Figure 7a), respectively. *L. mucosae* (9.20%) and *L. fermentum* (7.22%) in the CS group were approximately three times and two times, respectively, greater than in the SVD group, while the low abundance of *L. crispatus* (3.49%) in the SVD group was approximately 16 times higher than in the CS group. The dominant species in BF, MPF, and MF groups were *L. rhamnosus* and *L. gasseri*, which accounted for 70%, 60%, and 70% of the total lactobacilli, respectively (Figure 7b). In the BF group, *L. mucosae* (2.57%) was significantly lower, while *L. salivarius* (7.31%) was significantly higher than in the other groups. In the MPF group, although the high-abundance species *L. gasseri* was about 10% lower, low-abundance species such as *L. johnsonii* (4.20%), *L. delbrueckii* subsp. *bulgaricus* (3.30%), and *L. iners* (2.79%) were significantly higher than in the other groups.

#### 2.4.2. *Lactobacillus* Diversity and Related Factors Analysis in Early Infants

The difference in the samples between the groups was examined by principal component analysis (PCA) using the content distribution of different groups of *Lactobacillus*. The distribution of lactobacilli with different delivery modes showed that their distribution in both groups was similar, and there was no obvious clustering (Figure S4). The distribution of samples under different feeding methods showed that the dispersion of three groups was similar (Figure S4); only the individual samples in BF and MPF groups were scattered, and there was no obvious clustering among the three groups. In both factors, there was no significant difference, as the *p*-values of all indexes were greater than 0.05.

### 2.5. Functional Gene Composition of Gut Microbiota 

**Figure 8:**
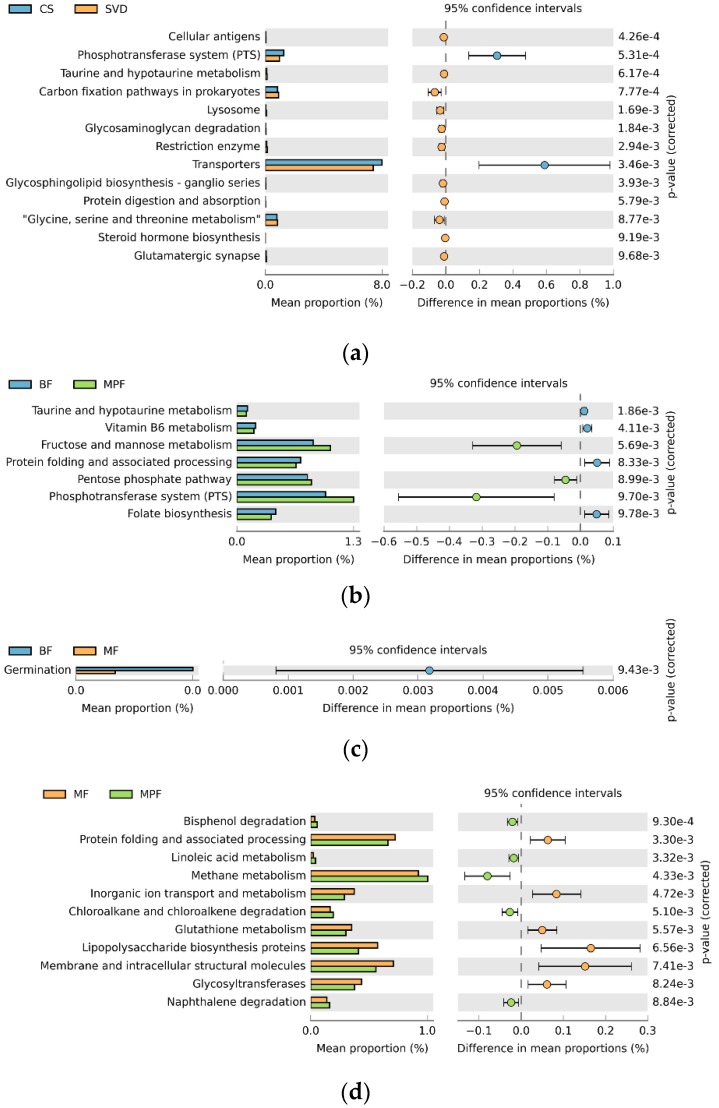
PICURSt function prediction of infant gut microbiota in different delivery modes and feeding methods. (**a**) Delivery modes; (**b**,**c**,**d**) Feeding methods.

To explore the effects of different delivery and feeding modes on physiological function, the Phylogenetic Investigation of Communities by Reconstruction of Unobserved States (PICURSt) software was used to predict and analyze the functional gene composition in the metabolic pathway. In delivery mode, the relative abundance of the 13 sub-functions of the secondary function prediction was significantly different. The functional genes of the phosphotransferase system (PTS) and transporters were significantly higher in the CS group, while 11 other functional genes such as those involved with cellular antigens and taurine and hypotaurine metabolism were higher in the SVD group (Figure 8a, *p* < 0.01). Comparing the three feeding methods in pairs showed that the relative abundances of seven sub-functions in the BF and MPF groups were significantly different. The functional genes of fructose and mannose metabolism, pentose phosphate pathway, and phosphotransferase system were significantly higher in the MPF group, while the functional genes of taurine and hypotaurine metabolism, vitamin B_6_ metabolism, protein folding and associated processing, and folate biosynthesis were higher in the BF group (Figure 8b, *p* < 0.01). In BF and MF groups, only the functional genes of germination were significantly higher in the BF group. There were significant differences in the relative abundance of 13 sub-functions between MF and MPF groups (Figure 8c, *p* < 0.01). In the MF group, six functional genes involved with protein folding and associated processing and inorganic ion transport and metabolism were significantly higher, while in the MPF group, seven functional genes such as those involved with bisphenol degradation and linoleic acid metabolism were higher (Figure 8d, *p* < 0.01).

## 3. Discussion

The establishment of infant gut microbiota is a complex process influenced by many factors, such as delivery mode, feeding, medication use, hospital environment, other early life experiences, and host genetics. This study focused on the effects of delivery and feeding methods on gut microbiota, at the level of phylum and genus, and at species level for *Bifidobacterium* and *Lactobacillus*.

At the phylum level, the dominant phyla among all the infants were Firmicutes and Proteobacteria. This is in agreement with earlier studies [16]. At the genus level, unclassified Enterobacteriaceae and *Bifidobacterium* were the common genera of both delivery modes, while SVD infants had more *Streptococcus* and *Bacteroides*, and CS infants contained more *Enterococcus* and *Clostridium*, with significant differences. There were also some low-abundance genera displaying significant differences, such as *Parabacteroides* and *Serratia*. While these were present in low abundance, their functions and correlations with diseases after adulthood need further study. Genera in SVD infant guts were more diverse, which might be because these infants had contact with maternal vaginal microorganisms at birth, while CS infants were exposed to the sterilized mother’s skin. Earlier studies reported that the diversity of gut microbiota in SVD infants after delivery was greater than CS [17]. Diversity in microbiota gradually converted with the onset of breastfeeding, as shown in previous reports [18]. This brings about an interesting question regarding the long-term effects of delivery mode and feeding methods, when the difference in gut microbiota was only observed in the first few months. The distribution of samples in different delivery modes showed that the CS group was separated from the SVD group, but they were clustered into different types, indicating that the two delivery modes had different effects on gut microbiota. The distribution of samples under different feeding methods showed that BF and MPF groups could be clustered into two types, while some samples of the MF group were dispersed in the other two groups, and only half of the samples were grouped together, indicating that breastfeeding and formula feeding had significant effects on gut microbiota, while mixed feeding had less of an effect on the difference in gut microbiota than the other two feeding methods. All the diversity results demonstrated that both delivery modes and feeding methods had a significant impact on the differences in gut microbiota.

The most abundant fecal microbiota with different feeding methods was unclassified *Enterobacteriaceae*, followed by *Bifidobacterium*, and BF infants were significantly higher in the abundance of these two bacteria than the other feeding methods. Among them, BF was also higher in the content of *Streptococcus*, while MPF resulted in significantly more *Enterococcus*, and MF infants had more *Clostridium*. This supports the earlier suggestion that SVD and BF are more conducive to the establishment of *Bifidobacterium* [19], suggesting a difference in the microenvironment in infants with different delivery and feeding methods, which may persist longer in life than the dietary effects. 

Numerous earlier publications reported on infant gut microbiota at phylum and genus level in different delivery and feeding methods [7], and some studies reported on the development of *Bifidobacterium* or *Lactobacillus* under different influencing factors [20]. We further extended the study to include the effects of different delivery and feeding methods on the composition and clustering of gut microbiota at the species level of *Bifidobacterium* and *Lactobacillus*. Analysis of the composition of *Bifidobacterium* found that the content of *B. animalis* subsp. *lactis*, *B. dentium*, and *B. longum* subsp. *infantis* were significantly different in delivery mode, possibly associated with the species of *Bifidobacterium* in the maternal genital microbiota, as suggested in an earlier literature [21]. The content of *B. dentium* in BF infants was significantly higher than in the other two feeding methods, which might be due to the presence of more *B. dentium* in the mother’s skin, transmitted to infants during feeding [22]. The Observed Species and Shannon index showed that there was a significant difference in describing different feeding methods, while in delivery mode, the *p*-value of all indexes were greater than 0.05 (i.e., the difference was not significant), indicating that the feeding methods had a significant effect on the difference of gut *Bifidobacterium*. Overall, the diversity of gut *Bifidobacterium* in early SVD and BF infants was higher, which is possibly a reflection of the varieties of human milk oligosaccharides (HMOs) supporting the various *Bifidobacterium* species [23]. For instance, the most common species recovered from infants (*B*. *bifidum*, *B*. *longum*, and *B*. *breve*) show vigorous or moderate growth on HMOs as a sole carbon source, while other strains such as *B. adolescentis* and *B. animalis* show no growth [24]. Further, α-1,2-fucosylated HMOs have been shown to promote the growth of some bifidobacteria, especially *B. longum*, *B*. *bifidum*, and *B. breve*, as these strains possess glycosyl hydrolase family 95 (GH95) or GH29 fucosidases that can hydrolyze 2-fucosylated HMOs [25].

In the composition of *Lactobacillus*, the content of *L. crispatus* in SVD infants was significantly higher than in the CS group, which might be related to the large number of *L. crispatus* in the mother's reproductive tract [26]. Simultaneously, the content of *L. mucosae* in BF infants was relatively more abundant, probably because the mothers’ skin was populated by more *L. mucosae* [27]. Moreover, the lactobacilli in CS and MPF infants were more diverse, which was different from the case of bifidobacteria. The varieties of lactose derivatives after heat treatment and sugars added in some cases in milk powder may have promoted the diversity of lactobacilli. Overall, the content of lactobacilli was less than that of bifidobacteria in SVD and BF infant guts, similar to previous report [28]. Further analysis of the differences in the effects of delivery and feeding methods on *Bifidobacterium* and *Lactobacillus*, the results showed that only feeding methods had a significant effect on the differences in the alpha diversity of *Bifidobacterium*, while there were no significant differences in *Lactobacillus* with different delivery and feeding methods, which was not consistent with previously reports [29].

Functional predictions showed that gut microbiota may have certain effects on the physiological functions of the host. In the SVD group, the functional genes of taurine and hypotaurine metabolism were significantly higher. Taurine combines with bile acid to form taurocholic acid, involved in the fat emulsification process, aiding in the digestion of mother’s milk fat. Studies have reported that *B. dentium* was more resistant to acid and bile salts [30]. This could further explain the abundance of *B. dentium* in BF infants. Cellular antigens are involved in the body’s immunity. *L. crispatus* has been reported to possess immunomodulatory effects [16]. It may play an important role in the maturation of immunity. 

The functional genes of PTS were higher in the CS group, which is involved in the glycolysis process. It was also found that the contents of *B. longum subsp. infantis*, *L. mucosae*, and *L. fermentum* were higher in the CS group. Among them, the PTS in *B. longum subsp. infantis* plays a role in the catabolic metabolism of sugars [31]. *L. mucosae* has a complete glycogen metabolism pathway in the pan-genome [32]. Sucrose-PTS and mannose-PTS in *L. fermentum* have a strong ability to metabolize glycogen [33]. As a whole, these bifidobacterial species in CS infants appear to enrich for the extraction of carbon and energy from glycogen and other carbohydrates. 

Breast milk and formula milk have different macronutrient composition. Most of the protein in formula milk is in the form of casein (78%), which forms large aggregates, and is not easily digested and absorbed. It has been reported to cause constipation, while breast milk only contains 40% casein [34]. The fatty acids contained in breast milk fat are unsaturated and dispersible in aqueous phase at body temperature, while formula milk has more saturated fatty acids, and linoleic acid—an important component of human brain cells—is deficient in formula milk [35]. 

This study also found that the functional genes of taurine and hypotaurine metabolism, vitamin B_6_ metabolism, protein folding and associated processing, and folate biosynthesis were significantly higher in the BF group. They are related to the metabolic pathways of fat and protein, and this could be explained by the presence of more digestible protein and unsaturated fatty acids in breast milk, thus contributing to the digestion and absorption process. 

In the MPF group, the functional genes of fructose and mannose metabolism, pentose phosphate pathway, and PTS were higher, which are related to the digestion and absorption of carbohydrates in the body, and might be due to the higher content of added sugar compared to lactose in formula. Simultaneously, the content of *B. adolescentis* and *L. johnsonii* was higher, and their ability to utilize mannose is stronger [36]. Therefore, this might be due to the differences in nutrients that lead to the differences in the relevant gut microbiota.

In summary, difference in the species level of *Bifidobacterium* and *Lactobacillus* was observed within the first 6 weeks, and might persist into adulthood leading to their lifelong effects. Modulation of gut environment and microbiota profile—particularly that of *Bifidobacterium* and *Lactobacillus* species—might be feasible approaches for remediation of the ill effects of CS and MPF on gut microbiota when they are unavoidable.

## 4. Materials and Methods 

### 4.1. Participants and Sample Collection

Fecal samples were collected from infant adsorbent nappies. To control the potential variants, fecal samples were randomly chosen as follow: (1) a balanced number of SVD and CS infants, (2) a balanced number of BF, MPF and MF infants from SVD and CS available samples. Additionally, technical criteria included the availability of >1 g of starting fecal material. Totally 112 Han ethnicity infants’ feces were collected, in which 44 were from SVD infants and 68 were from CS infants; 41 were BF infants; 39 were MPF infants and 32 infants were MF. And 62 of those 112 infants were girls (55%). For the definition of feeding methods, the BF group consisted of infants receiving breast milk as the sole source of milk; the MPF group included those infants receiving some human milk and some milk powder; the MF group consisted of those infants receiving infant formula as the sole source of milk. The gestational age of those infants was 39.7 ± 0.05 weeks. There were no significant differences in infant gestational age, birth weight, weight at sampling, etc., except for delivery mode and feeding methods. The infants’ body weight at birth were 3376 ± 232g, with a weight for age z-score of 0.87 ± 0.12, while the infants’ body weight at sampling were 4254 ± 385g, with a weight for age z-score of 0.92 ± 0.07. The age of infants at sampling was 41.53 ± 1.15 days after birth. The study was approved by the Ethical Committee of Jiangnan University (approved on 3 November 2016).

### 4.2. Fecal Genomic DNA Extraction

Genomic DNA was extracted from 0.1 g stool samples using FastDNA^®^ Spin Kit (MP Biomedicals, Santa Ana, CA, USA) following the instructions. DNA was visualized on a 1% agarose gel and quantified by ND-2000 spectrophotometer (Nanodrop Inc., Wilmington, DE, USA), and stored at −80 °C.

### 4.3. DNA Amplification

The V3–V4 region was amplified using primers 341F (5′-CCTAYGGGRBGCASCAG-3′) and 806R (5′-GGACTACNNGGGTATCTAAT-3′), as previously described [37]. The PCR amplification was carried out with the 2×Taq MasterMix (CWBIO, Beijing, China) following the manuals. The amplification consisted of an initial denaturation at 95 °C for 5 min, followed by 30 cycles, where 1 cycle consisted of 95 °C for 30 s, 52 °C for 30 s, and 72 °C for 30 s, and a final extension of 72 °C for 7 min. 

Assessment of *Bifidobacterium* species was carried out as described previously [38]. Bifidobacterial *groEL* gene (*Bif-GroEL*) was amplified using primers *Bif-groEL*-F (5′-TCCGATTACGAYCGYGAGAAGCT-3′) and *Bif-groEL*-R (5′-CSGCYTCGGTSGTCAGGAACAG-3′). The PCR amplification was carried out with the Premix Taq^TM^ (TaKaRa, Dalian, China) following the manuals. The PCR procedures were pre-denatured at 95 °C for 5 min, followed by 30 cycles consisting of denaturation at 95 °C for 30 s, annealing at 60 °C for 30 s, and extension at 72 °C for 1 min, and a final extension of 72 °C for 10 min.

Assessment of *Lactobacillus* species was carried out following a similar protocol as *Bifidobacterium*. *Lactobacillus groEL* gene (*Lac-GroEL*) was amplified using primers *Lac-groEL*-F (5′-TCCGATTACGAYCGYGAGAAGCT-3′) and *Lac-groEL*-R (5′-CSGCYTCGGTSGTCAGGAACAG-3′). The amplification procedures included a pre-denaturation step at 95 °C for 5 min, followed by 35 cycles consisting of denaturation at 95 °C for 30 s, annealing at 58 °C for 30 s, and extension at 72 °C for 1 min, and a final extra extension at 72 °C for 7 min. 

### 4.4. Library Preparation and Sequencing

All the PCR products were excised from a 1.5% agarose gel, purified by a QIAquick Gel Extraction Kit (QIAGEN, Hilden, Germany) and quantitated using the Qubit^TM^ dsDNA BR Assay Kit (Life Technologies, Carlsbad, CA, USA) according to the manuals. DNA amplicon libraries were prepared by TruSeq Nano DNA LT Kit (Illumina, San Diego, CA, USA) and sequenced with the MiSeq Reagent Kit v3 (600 cycles-PE, Illumina, San Diego, CA, USA) on the MiSeq Illumina platform following the instructions.

### 4.5. Bioinformatics Analysis

The sequence reads were processed with QIIME (Quantitative Insights Into Microbial Ecology) package (Flagstaff, AZ). The raw reads were screened as previously described [39]. Only pair-end reads overlapping >10 bp and without any mismatch were assembled. Barcode and sequencing primers from the above assembled sequences were trimmed. The operational taxonomic units (OTUs) were established de novo using UCLUST with a 97% sequence identity cut off. The OTUs of the V3–V4 region were assigned by the Ribosomal Database Project (RDP) Naive Bayes classifier. The OTUs of *Bif-GroEL* sequences and *Lac-GroEL* sequences were assigned by *Bifidobacterium groEL* database and *Lactobacillus groEL* database, respectively.

The Python Nearest Alignment Space Termination (PyNAST) aligner was used to compare the sequence with the Greengenes core set [40]. The phylogenetic tree was generated by FastTree [41], and the dilution curve was drawn to calculate the alpha diversity and beta diversity of the sample performed by QIIME. The similarities among the microbial communities were estimated using the principal coordinate analysis (PCoA) relying on unweighted and weighted UniFrac.

Chimeric sequences were detected and removed based on the “RDP Gold” database using UCHIM software [42]. The OTU cluster analysis was performed according to the Galaxy online platform process [43]. The BIOM file obtained by the QIIME software was uploaded to the Galaxy website for predictive analysis of the Phylogenetic Investigation of Communities by Reconstruction of Unobserved States (PICURSt) functional genes [44]. The information could be obtained by referring to the Kyoto Encyclopedia of Genes and Genomes (KEGG) Orthology class 1 and class 2 functional gene classes to obtain the functional composition of the predicted genome.

### 4.6. Statistical Analysis

Significant differences among different groups were judged by ANOVA, and the differences between the two groups were judged by *t*-test (SPSS 16.0). *p* < 0.05 was considered to be significant. 

## Supplementary Materials

It can be found at: https://www.mdpi.com/1422-0067/20/13/3306/s1.

## Abbreviations

BF	Breast-Fed
*Bif-GroEL*	Bifidobacterial *groEL* Gene
CS	Caesarean Section
HMOs	Human milk oligosaccharides
KEGG	Kyoto Encyclopedia of Genes and Genomes
*Lac-GroEL*	Lactobacillus *groEL* Gene
LDA	Linear Discriminant Analysis
LEfSe	Linear discriminant analysis effect size
MF	Mixed-Fed
MPF	Milk-Powder-Fed
OTU	Operational Taxonomic Unit
PICURSt	Phylogenetic Investigation of Communities by Reconstruction of Unobserved States
PCA	Principal component analysis
PCoA	Principal coordinate analysis
PTS	Phosphotransferase System
PyNAST	Python Nearest Alignment Space Termination
QIIME	Quantitative Insights into Microbial Ecology
RDP	Ribosomal Database Project
SVD	Standard Vaginally Delivered
