# Supplementary material for: Bifidobacterium and Lactobacillus Composition at Species Level and Gut Microbiota Diversity in Infants before 6 Weeks
Source: Int J Mol Sci. 2019 Jul 5;20(13):3306. doi: 10.3390/ijms20133306 (PMC6650860; doi:10.3390/ijms20133306)
Supplement: Supplementary file 1 [file ijms-20-03306-s001.pdf]

International Journal of
Molecular Sciences

MDPI

Supplementary Materials

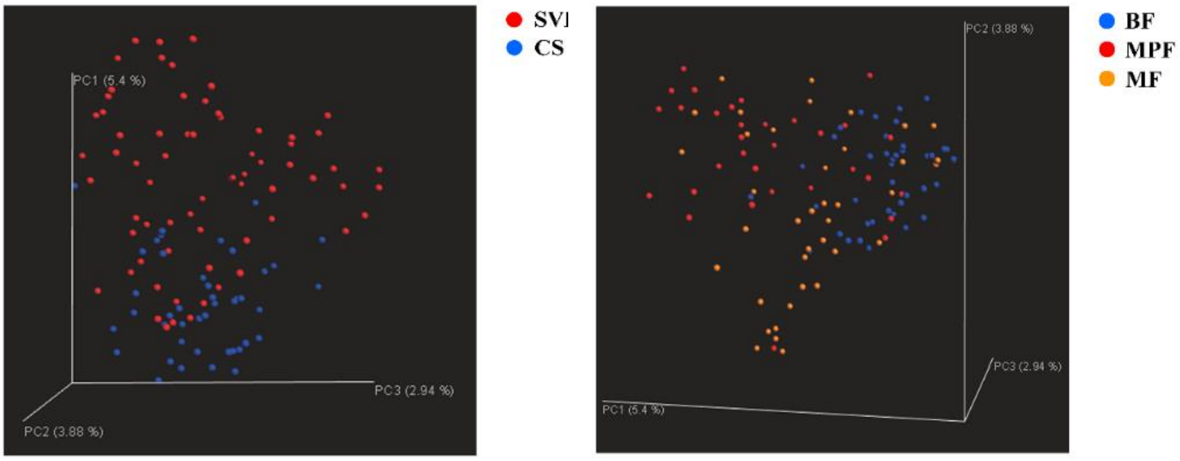

(a) **(b)**

Figure S1. PCA of gut microbiota in the infants with different delivery modes and feeding methods. (**a**): delivery modes; (**b**): feeding methods.

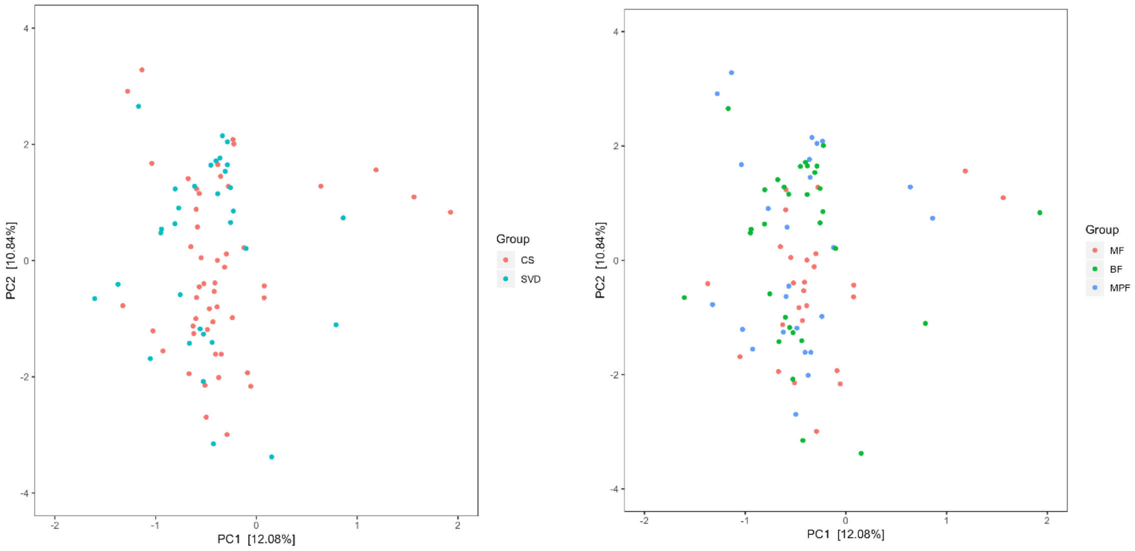

(**a**) (**b**)

Figure S2. PCA of gut *Bifidobacteria* community in the infants with different delivery modes and feeding methods. (**a**): Delivery modes; (**b**): Feeding methods.

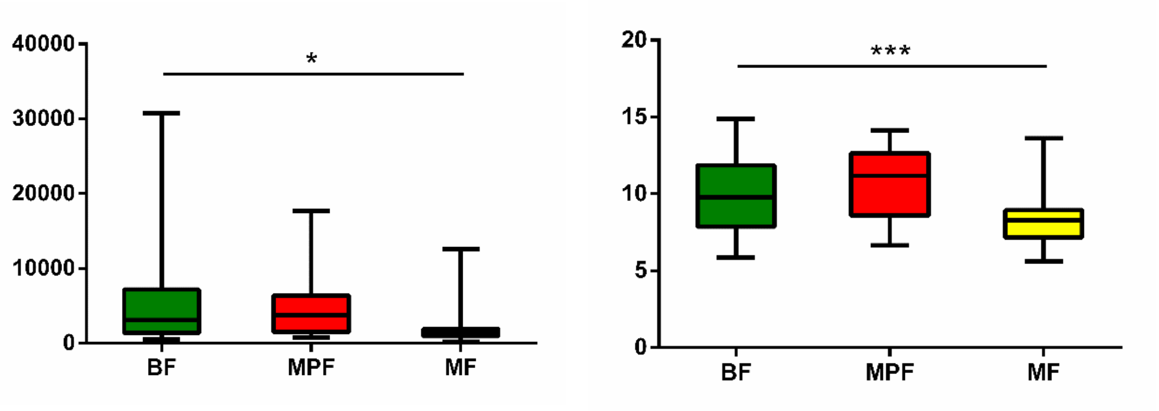

(**a**) (**b**)

Figure S3. Alpha diversity analysis of gut *Bifidobacteria* community in the infants with different feeding methods. (**a**): Observed species ($p < 0.05$); (**b**): Shannon ($p < 0.001$). *$p < 0.05$, ***$p < 0.001$.

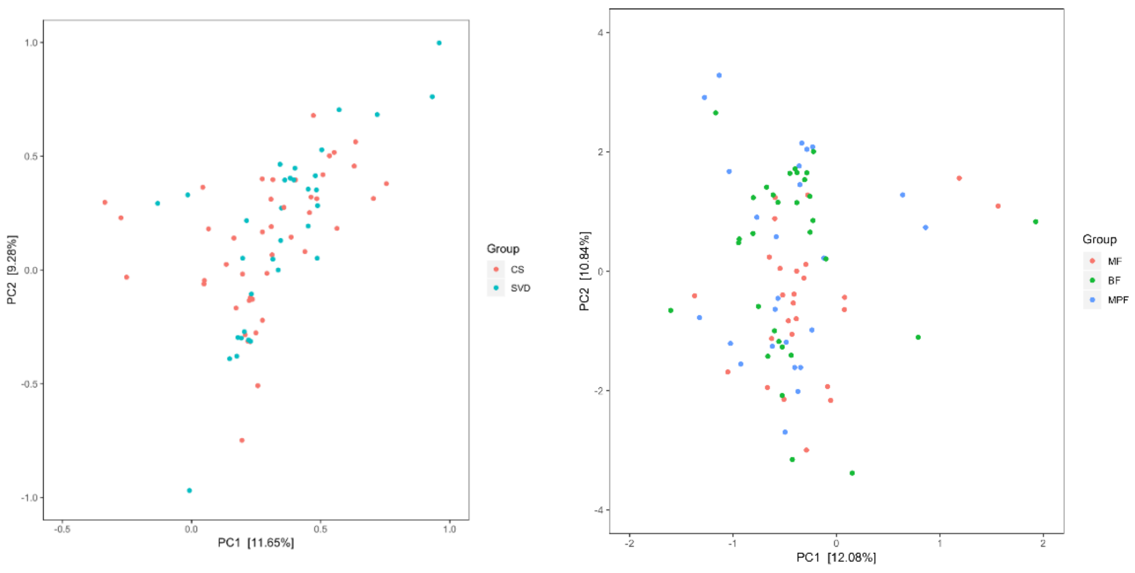

(**a**) (**b**)

Figure S4. PCA of gut *Lactobacillus* community in the infants with different delivery modes and feeding methods. (**a**): Delivery modes; (**b**): Feeding methods.
